# Does Mothers’ Self-Construal Contribute to Parenting Beyond Socioeconomic Status and Maternal Efficacy? an Exploratory Study of Turkish Mothers

**DOI:** 10.3389/fpsyg.2018.01245

**Published:** 2018-07-24

**Authors:** Feyza Corapci, Hande Benveniste, Sibel Bilge

**Affiliations:** ^1^Psychology Department, Boğaziçi University, Istanbul, Turkey; ^2^Psychology Department, Okan University, Istanbul, Turkey

**Keywords:** autonomous-related, self-construal, parenting, maternal efficacy, socioeconomic status

## Abstract

This study examined the relative contribution of mothers’ self-construal to parenting above and beyond family socioeconomic status (SES) and maternal efficacy beliefs about parenting. A total of 58 Turkish mothers and their preschool-aged children participated in dyadic tasks in the laboratory setting. For the measurement of parenting, direct behavioral observations of mother–child interactions in three interaction contexts were utilized, and mother ratings of emotion socialization were obtained. Mothers also reported on their parenting efficacy, self-construal, child temperament, and family demographics. Results revealed a more balanced endorsement of autonomous and relational self-characteristics as well as more sensitive parenting among higher SES mothers. Furthermore, mothers’ self-construal contributed unique variance to the prediction of sensitive parenting over and above SES, maternal efficacy and child temperament. Yet, in the prediction of negatively controlling parenting, mothers’ self-construal did not account for unique variance. Lower parenting efficacy and lower SES were the only predictors of punishing, overriding, and distress magnifying responses. Finally, results indicated a marginally significant indirect effect from SES to sensitive parenting via autonomous-related self-construal, controlling for the indirect effect of maternal efficacy.

## Introduction

Parenting is a key mechanism of child socialization (Kochanska, 1997). The quest for factors that contribute to variations in parenting has intrigued researchers to better understand child socialization. Belsky’s (1984) model of the determinants of parenting in general and Grolnick’s (2002) model of autonomy-supportive versus controlling parenting in particular, identify child characteristics, mothers’ resources in caregiving, and social context as crucial predictors of parenting. Research with ethnic and cultural groups has also drawn attention to the role of cultural values and beliefs on parenting (Trommsdorff and Cole, 2011). In particular, self-construal, defined as a “constellation of thoughts, feelings, and actions concerning one’s relationship to others” (Singelis, 1994, p. 581), is believed to reflect cultural values about autonomy- and relatedness-orientations at the individual level (Dennis et al., 2002). However, there is paucity of research on the role of culturally-mediated self-construal on parenting although self-construal has been related to a variety of outcomes such as information processing, motivation, and interpersonal responses in peer or romantic relationships (see a review by Cross et al., 2011).

We aimed to extend past research by considering mothers’ self-construal as a predictor of parenting within the context of urban Turkish culture, where the cultural model of self-construal is theorized as autonomous-related, i.e., individuals organize autonomous acts around relationship with close others (Kagitcibasi, 2007). Integrating Belsky’s parenting model with Kagitcibasi’s self-theory, we examined the relative contribution of mothers’ self-construal to parenting practices above and beyond the family socioeconomic status (SES) and mothers’ caregiving efficacy by using direct behavioral observations of mother–child interactions in the laboratory setting and by utilizing mother ratings about emotion socialization.

## Sensitive Parenting Practices in Early Childhood

Starting in the first year of life and extending to later years, children need to feel close, safe and connected with their caregivers (Carlson et al., 2004). In toddlerhood and preschool period, important and rapid changes in locomotor, socio-emotional and cognitive abilities foster the foundation of independence and self-governance. Consequently, children have an increased awareness of themselves as separate from others, which motivates them to experience self-sufficiency, exert personal choices in various personal daily activities, and seek an autonomous sense of self (Mascolo and Fisher, 2007).

Caregivers’ behavioral and affective qualities that serve to meet the needs of children for relatedness, autonomy, and competence are accepted as sensitive parenting practices based on developmental theory and research (Vereijken et al., 1997; Dix, 2000; Carlson et al., 2004; Soenens et al., 2017). In particular, caregivers’ supportive and affectionate presence as well as autonomy granting are parenting practices that act to support children’s needs for protection, comfort, assistance, and independence as well as exploration, respectively (Carlson et al., 2004). Specifically, caregivers are considered supportive when they correctly interpret and appropriately respond to children’s cues and share positive affect with them (Biringen et al., 2000; Carlson et al., 2004; Kochanska and Aksan, 2004). A crucial complement to supportive parenting is caregivers’ respect for autonomy that involves validating the child’s perspective, suggesting ideas to foster volitional behavior, intervening when asked for assistance, and following the child’s lead whenever possible (Grolnick, 2002; Sroufe et al., 2009).

Most previous research has combined supportive presence and autonomy granting into a sensitive parenting composite based on conceptual and empirical grounds (Kochanska, 1997; Jaffari-Bimmel et al., 2006; Bell and Belsky, 2008; Burchinal et al., 2014). Research has consistently shown that sensitive parenting relates to children’s secure attachment relations, self-regulation skills, mastery motivation and cognitive competence (Renken et al., 1989; Denham et al., 2007; Bindman et al., 2015; Lucassen et al., 2015).

Socialization also aims at fostering emotional competence and involves caregivers’ specific reactions in response to child’s anger, sadness, and fear (Denham et al., 2007). Emotion socialization, although embedded within the broader parenting practices, is distinguished by its circumscribed process and largely constrained to teaching children about understanding, expressing and regulating emotions (Denham et al., 2007). Research shows that caregivers who are aware of and accept their own and their child’s negative emotions are more likely to provide rewarding experiences that involve showing empathy, comforting, and scaffolding. On the other hand, caregivers who consider emotions as experiences to be avoided, are more likely to neglect or override the child’s emotional experiences by minimizing or diverting child’s attention from the emotion. Finally, caregivers who consider emotions as unacceptable, may themselves get distressed, exert control by punitive responses, and magnify the child’s negative emotions (O’Neal and Magai, 2005; Denham et al., 2007). In considering the influence of emotion socialization, growing research indicates the positive role of rewarding experiences on children’s emotional and social competence, whereas non-supportive ways of responding to children’s emotions (e.g., punishing, minimizing) were related to poorer socioemotional adjustment (Denham et al., 2007).

## Distal and Proximal Predictors of Parenting

In Belsky’s seminal model, the multiply determined nature of parenting practices is highlighted with regard to the contextual and caregivers’ stressors and resources as well as child characteristics. Below we review the role of family SES as a contextual factor on parenting followed by the role of maternal efficacy as reflecting mothers’ personal resources.

### Socioeconomic Status (SES)

Family SES, often conceptualized as an index of family income, parental education and occupational status, is considered as a distal yet one of the most crucial predictors of parenting (Bornstein and Bradley, 2002; Evans et al., 2008). Given the associated demographic, psychosocial and contextual risk factors, socioeconomic disadvantage is expected to diminish caregivers’ capacity for being sensitive to children’s needs for relatedness and autonomy (Bornstein and Bradley, 2002). Indeed, evidence from longitudinal (El-Sheikh et al., 2010) and cross-sectional research (Zevalkink and Riksen-Walraven, 2001; Berlin et al., 2002; Ispa et al., 2004) show that mothers from lower SES families are more likely act in non-supportive, intrusive as well as controlling ways toward their children.

### Maternal Efficacy

As mothers need to manage complex tasks in caregiving and balance the needs of their children with the needs and demands of the family context, personal psychological resources are seen as key proximal predictors of parenting. One resource is maternal efficacy that refers to mothers’ perceived competence to manage various tasks of caregiving (Teti and Gelfand, 1991). Thus, maternal efficacy involves domain-specific beliefs in caregiving and results from a history of perceived mastery experiences in caregiving tasks (Jones and Prinz, 2005). Comprehensive reviews have concluded that high levels of maternal efficacy relate to mothers’ responsiveness to child signals, the degree of warmth toward the child and effective limit setting, even after controlling for social support and child temperament (Teti and Gelfand, 1991; Jones and Prinz, 2005).

## Self and Family Models in the Global Contextual Change

Self-construal, which refers to how separate or connected one feels in relation to others is another individual-level, proximal factor of social behavior (Markus and Kitayama, 2010). Yet, we know little about how mothers’ self-construal relates to parenting. To fill in this gap in research, our study was guided by Kagitcibasi’s (2007) self and family model framework.

In this framework, self-construal is conceptualized as embedded within the family, which in return, is situated within the larger sociocultural system with its sociodemographic features and culturally shared values (Kagitcibasi, 2007). Accordingly, as individualistic, urban, and industrialized Western societies prioritize self-reliance, assertiveness, and personal needs, these values are reflected in the prevailing *autonomous-separate* self: individuals define themselves as agentic with clearly defined self-boundaries, who organize self-directed acts primarily around personal goals. This view about the nature of self also acts to influence the relative emphasis parents place on individualistic and relational socialization goals and practices (Trommsdorff and Cole, 2011).

On the other hand, agricultural and rural societies endorsing collectivism prioritize group interests over individual goals. Predominant values stress fulfilling one’s obligations and adhering to social norms. These values are reflected in the *related* self-construal such that individuals define themselves by social responsibilities and perceive permeable self-boundaries that overlap with those of others (Kagitcibasi, 2007). Obedience is a highly valued child socialization goal given the relative importance of group needs over personal goals. Thus, to instill socially appropriate acts, caregivers foster restraint of behaviors and emotions that can be disruptive to interpersonal relations, primarily by obedience-demanding discipline (Raval et al., 2012).

With sociodemographic changes in the global context, a cultural shift toward autonomy and assertiveness emerged among highly educated, urban families in developing collectivistic societies like India, China, Japan, and Turkey (Kagitcibasi and Ataca, 2005; Mayer et al., 2012; Raval et al., 2012; Okur and Corapci, 2015). Kagitcibasi (2007) proposed that a dialectic synthesis of individual and group needs in developing collectivist societies would foster the development of *autonomous-related* selves: individuals who can balance self-directed and assertive acts within the context of close-knit relations to adapt to the competitive urban life.

Child socialization in these fast-developing collectivist societies draws on sensitive caregiving that entails warmth, explanations for proper behavior, and growing respect for autonomy (Raval et al., 2012). Increasing support for child’s agency is also reflected by mothers’ increasing preference for autonomy-oriented socialization goals (Keller et al., 2006). For instance, middle-class, educated Indian mothers endorse scolding less often than traditional Indian mothers, yet endorse comforting responses to their distressed child at similar rates as US mothers (Raval et al., 2012).

## Cultural Context of Turkish Families

Characterized as a collectivistic culture (Hofstede, 2001), Turkish society is undergoing major social change due to industrialization and urbanization. In support of Kagitcibasi’s theoretical premise, research shows that self-descriptions of young Turks include both individualist and collectivist values (Imamoğlu and Karakitapoğlu-Aygün, 2007). Studies with Turkish college students have also revealed that they endorse more autonomous-related self-construal than non-students (Kagitcibasi et al., 2006), and describe themselves as autonomous as Belgian students, but more related to their mothers than their Belgian counterparts (Coskan et al., 2016).

When socializing children, autonomy-oriented socialization goals and practices are endorsed more, whereas punitive, controlling and obedience-demanding practices are endorsed less by higher SES urban Turkish mothers than their lower SES counterparts (Kagitcibasi and Ataca, 2005; Nacak et al., 2011; Mayer et al., 2012). Naturalistic home observations also reveal that higher SES Turkish mothers of 3-year-old children are less likely to display controlling (e.g., verbally or physically intruding the child) and non-responsive behaviors than lower SES mothers (Akcinar and Baydar, 2014; Baydar and Akcinar, 2015). A recent study on emotion socialization has revealed that middle-high SES Turkish mothers endorse encouragment of the child for independent coping along with comforting and reasoning responses, suggestive of a combination of autonomy- and relatedness-orientation (Corapci et al., 2018). These results suggest that higher SES Turkish mothers convey messages about the autonomous-related self in socializing children. Yet, the unique association of mothers’ self-construal with parenting beyond the contribution of family SES and maternal efficacy remains to be investigated.

## Present Study

The present study aimed to extend the literature in a number of ways. First, given the diversity among urban families in terms of the affluence and education level, we conducted an intracultural assessment of Turkish mothers’ self-construal. Mothers of higher SES were expected to endorse more autonomous-related self-characteristics compared to lower SES mothers as this self-construal model is theorized to emerge with socioeconomic growth in developing collectivist cultures.

Next, we expected mothers with more balanced autonomous and relational self-characteristics to engage in more sensitive parenting above and beyond the contribution of SES and maternal efficacy. On the other hand, mothers’ autonomous-related self-construal was expected to relate negatively to non-supportive responses. Finally, we hypothesized that mothers’ autonomous-related self and possibly maternal efficacy would mediate the relation between family SES and mothers’ parenting practices. When testing these study hypotheses, the potentially confounding role of child temperament was also examined.

Finally, although the focus of the present study with urban Turkish mothers was on the cultural model of autonomous-related self-construal, the role of other types of self-construal was also explored. Based on Kagitcibasi’s (2007) model, we predicted a negative association between SES and mothers’ related self. Related self was expected to relate negatively to sensitive parenting and positively to non-supportive parenting. No association was expected between autonomous-separate self and SES as well as parenting within the context of Turkish culture.

## Materials and Methods

### Participants

Participants consisted of 58 Turkish mothers and their children (50% boys). Child age ranged between 4 and 6 years (*M* = 57.14 months, *SD* = 7.56). Mothers’ mean age was 37.25 years (*SD* = 5.18). Maternal and paternal education level was rated on a 10-point scale (1 = *less than primary school* to 10 = *graduate degree*). Of the mothers, 21% had less than a high school degree, 19% were high school graduates, 27% had college education, and 33% had a graduate degree. Paternal education distribution was similar: 19% had less than a high school degree, 15% graduated from high school, and 66% had at least college degree or higher. Sixty-nine percent of the mothers and 93% of the fathers were employed. Almost all children (98%) were from intact families. In the present study, an SES score was formed by taking the mean of standardized values for both parents’ educational levels and household income level.

### Procedure

This study was part of a larger study on preschoolers’ socioemotional development. Each mother–child dyad attended an approximately 1-h session in a research laboratory. For the present study, behavioral observations of mother–child interactions from three dyadic contexts were used. During the 5-min free play, each mother–child dyad was presented with various toys and instructed to play as they wish. During the 5-min snack time, the research assistant left a snack basket in the room and told the mother and child that they may have whatever snack and beverage they choose. The 5-min drawing task required each mother–child dyad to work cooperatively on a drawing activity using an Etch-a-Sketch board. The dyad was asked to draw a picture of a house presented to them as a template. In this task, the mother was instructed to use only one knob to draw horizontal lines, and the child was asked to use the other knob to draw vertical lines. All tasks were videotaped from behind a one-way mirror for later coding. To measure mothers’ responses to child’s negative emotions, a parent self-report measure was used. Mothers also completed additional surveys on their self-construal, caregiving efficacy, their child’s temperament, and family demographics.

### Measures

#### Maternal Supportive Presence and Autonomy Granting

The videotaped mother–child interactions were coded using the scales developed by Erickson et al. (1985) to rate maternal supportive presence and autonomy granting. Supportive presence was defined as mothers’ emotional support, positive regard, and recognition of child’s needs followed by prompt responses. On a seven-point scale, ratings from 1 to 3 describe inadequate supportive presence (i.e., mother either completely fails to provide support or provides limited support that is sporadic and poorly timed). A rating of four pertains to respectable yet inconsistent supportive presence, and a rating of five describes a mother with fairly adequate supportive presence who at times fails to modulate her support to the child’s needs. Ratings from 6 to 7 characterize very high levels of supportive presence (i.e., mother is supportive and encouraging throughout the session, reinforces the child’s success and modulates her responsiveness according to child’s needs).

Respect for child’s autonomy was also rated on a seven-point scale that evaluated the extent to which mothers validate child’s interests, preferences, intentions, and provide the child opportunities for self-initiated actions, and respect child’s motives in decision making. Ratings from 1 to 3 refer to high intrusiveness as reflected by a mother’s imposition of her own agenda, her physical interference and interruptions as well as disregard for child’s ideas. A rating of four describes a mother with moderate intrusiveness (i.e., mother does not deny child’s individuality but provides little encouragement to child’s expression of his/her own ideas and preferences and might communicate doubts about child’s individuality), whereas a rating of five pertains to moderate autonomy granting (i.e., mother engages the child by reflecting on his/her ideas but also exerts her own agenda). Ratings from 6 to 7 describe a mother who clearly trusts and acknowledges her child’s individuality and engages in mutually negotiated decision making.

In the present study, supportive presence and autonomy granting were coded by two different teams of two raters each to avoid inflated correlations between these two variables due to common rater bias. Coders were trained by the first author to an initial interrater reliability of intraclass correlation (ICC) ≤ 0.75 using practice tapes of previous recorded dyads. Ongoing interrater reliability was established on 20% of the cases that were independently coded by two coders. ICCs between raters, who were blind to study hypotheses, were 0.79 for supportive presence and 0.86 for autonomy granting.

#### Expression of Positive Emotions

During snack time, each mother–child dyad was seated at a table facing the camera. This allowed us to clearly view the faces to code not only the mother’s but also the dyad’s shared positive emotion expression during snack time. Positive emotion was coded based on facial, vocal, and behavioral cues such as smiles or laughter. In 5-s epochs, mother-only positive emotion was coded when the mother alone displayed positive emotion, whereas shared positive emotion was coded when both mother and child displayed co-occuring smiles or laughter. Proportion scores were computed, i.e., the number of epochs with mother-only and shared positive emotion coded as present divided by the total number of epochs. Coding was performed by an independent team of two coders who were trained to an initial interrater reliability (kappa) of 0.70. For ongoing reliability, randomly selected tapes were independently rated by two coders. Kappas ranged from 0.65 to 0.84.

#### Data Reduction

In the present study, behavioral ratings across three contexts were significantly correlated for supportive presence, ranging from 0.36 to 0.55 (*p*s < 0.01 to 0.001), and for autonomy granting, ranging from 0.43 to 0.59 (*p*s < 0.001). Relying on previous work (Kochanska and Aksan, 2004), supportive presence and autonomy granting scores were averaged across these contexts to form overall composites. Next, correlations between those overall composites for supportive presence and autonomy granting, and smile scores were computed. Based on the significant associations amongst these variables (*r*s ranged from 0.32 to 0.46, *p*s < 0.012 to 0.001), they were standardized and averaged to form an aggregated *sensitive parenting composite.* High scores on this composite indicated mothers who responded sensitively to the needs of their child and respected the child’s autonomy within an affectively positive interaction context.

#### Maternal Responses to Negative Emotions

Mothers completed the Responses to Children’s Emotions scale to report how they react to their child’s negative emotions. The original scale, developed by Klimes-Dougan et al. (2007) was adapted by Hastings and De (2008) to use with parents of preschoolers. The scale prompted mothers to think about times when their child has been angry, sad, or afraid over the past two months. To each emotion, mothers rated the likelihood of responding in 15 possible ways on a five-point Likert-type scale (1 = *not at all typical* to 5 = *very typical*). The scale has five response categories. Rewarding responses entail warmth and assistance (e.g., *I comforted my child*; *I found out what made my child angry*). Neglecting responses entail caregivers’ failure to notice or respond to child’s emotion (e.g., *I usually didn’t notice; I ignored my child*). Overriding responses dismiss the child’s emotional experience by minimizing or distracting child (e.g., *I told my child not to worry; I told my child to cheer up*). Punishing responses disapprove the child’s emotion by verbal or non-verbal ways (e.g., *I gave my child a disapproving look; I said he should be ashamed*). Magnifying responses reflect caregivers’ own distress at child’s emotion experience (e.g., *I got all upset*).

#### Data Reduction

All corresponding subscales to fear, sadness, and anger were positively and significantly correlated, *r*’s ranged from 0.54 to 0.68 for the Reward, from 0.27 to 0.42 for the Override, from 0.43 to 0.60 for the Neglect, from 0.27 to 0.42 for the Punish, and from 0.27 to 0.52 for the Magnify subscales, with *p*’s < 0.05 to 0.001. These subscales across three emotions were averaged to form aggregated subscale scores. Alphas ranged from 0.68 to 0.84. Next, these aggregated subscales were subjected to a principal components analysis with Varimax rotation to extract factors to reduce the number of variables and lessen the number of statistical tests. The Kaiser-Meyer Olkin measure revealed acceptable sampling adequacy, KMO = 0.70. A two-factor structure with eigenvalues larger than 1.0 was obtained that accounted for 74% of the variance. Override, Punish, and Magnify loaded on the first factor, with loadings of 0.74, 0.87, and 0.83, respectively. Reward and Neglect loaded on the second factor, with loadings of 0.85 and -0.62, respectively. These two factors were theoretically meaningful and interpretable to define non-supportive and supportive dimensions of emotion socialization. Thus, subscale scores on each factor were averaged to form composite scores, with reverse coded Neglect scores on the second factor.

#### Maternal Efficacy

Mothers’ beliefs on their caregiving efficacy was measured with the 11-item Sense of Incompetence subscale of the Parenting Stress Index (PSI; Abidin, 1997). Example items include “*I feel capable and on top of things when I am caring for my child*; *I often have the feeling that I cannot handle things very well*.” Mothers rated all items on a five-point Likert scale (1 = *strongly disagree* to 5 = *strongly agree*). Given that high scores on this measure indicate lower sense of competence, the total maternal efficacy score was reversed for ease of interpretation. Cronbach alpha of this subscale in the present study was 0.71. The mean score for this sample of mothers was 2.38 (*SD* = 0.52).

#### Mothers’ Self-Construal

Mothers completed the nine-item Autonomous-Related Self-Construal subscale (Kagitcibasi et al., 2006) to report how much they agreed with the compatibility of relatedness and autonomy (e.g., *A person may be attached to those who are close, and at the same time, expect respect for any differences of opinion*). High scores on this scale shows higher level of agreement with the coexistence of agency and relatedness. Mothers also completed the nine-item Related Self-Construal subscale (e.g., *The people who are close to me strongly influence my personality*), and the nine-item Autonomous-Separate Self subscale (e.g., *People who are close to me have little influence on my decisions*). Mothers rated each item using a five-point Likert type scale (*1 = totally disagree* to *5 = totally agree*). In previous research, Cronbach alphas for these subscales with college students were adequate, ranging from 0.74 to 0.84 (Kagitcibasi et al., 2006). The scale’s validity was demonstrated by higher autonomous-related self scores of university students compared to non-students who represented lower SES young adults (Kagitcibasi et al., 2006), and by the associations of autonomous-related self scores with adolescents’ psychological well-being, resilience and life satisfactions (Ozdemir, 2012; Yildirim et al., 2015). In the present study, Cronbach alphas were 0.67, 0.73, and 0.66 for the Autonomous-Related, Related, and Autonomous-Separate self subscales, respectively.

#### Child Temperament

The Child Behavior Questionnaire (CBQ; Goldsmith and Rothbart, 1991) is a temperament questionnaire for children 3 to 7 years of age. Caregivers rated how well each item applied to their child on a seven-point scale ranging from 1 (*extremely untrue of your child*) to 7 (*extremely true of your child*). Mothers completed the six-item Attention Shifting and the eight-item Attention Focusing subscales. These items pertain to children’s children’s ability to concentrate and maintain attentional focus on tasks at hand and shift focus when required. In the present study, Cronbach alphas for Attention Focusing and Shifting subscales were 0.65 and 0.61, respectively.

#### Family Demographics

Mothers reported about their age and child age, family size, marital status, family income, maternal and paternal education as well as occupation.

## Results

### Descriptive Statistics and Correlations Among Study Variables

Distributions of the variables were generally normal and no outliers were identified. Descriptive statistics and bivariate correlations between the parenting variables, maternal efficacy, SES and self-construal subscales are presented in **Table 1**. SES was positively related to the autonomous-related and related dimensions of mothers’ self-construal as well as observer ratings of sensitive parenting and negatively related to the non-supportive emotion socialization. SES was not associated with the autonomous-separate dimension of mothers’ self-construal. Maternal efficacy and the autonomous-related dimension of mothers’ self-construal were positively related to observer ratings of sensitive parenting and mother ratings of supportive emotion socialization, and inversely related to non-supportive emotion socialization. Maternal efficacy also showed a marginally positive relationship with SES (*p* = 0.054).

**Table 1:** Descriptive statistics and intercorrelations among study variables.

Variable	1	2	3	4	5	6	7	8
(1) Observed sensitive parenting	–							
(2) Supportive ES	0.42^∗∗^	–						
(3) Negatively controlling ES	–0.34^∗∗^	–0.12	–					
(4) SES	0.54^∗∗∗^	0.06	–0.56^∗∗∗^	–				
(5) Maternal efficacy	0.35^∗∗^	0.32^∗∗∗^	–0.50^∗∗∗^	–0.25^†^	–			
(6) Autonomous-related self	0.35^∗∗^	0.40^∗∗∗^	–0.30^∗^	0.42^∗∗^	–0.24^†^	–		
(7) Related self	0.14	0.22	–0.09	0.31^∗^	–0.03	0.21	–	
(8) Autonomous-separated self	0.17	0.09	–0.14	0.14	0.19	0.14	–0.58^∗∗∗^	–
Mean (SD)	0.00 (0.85)	1.52 (0.39)	2.74 (0.54)	0.00 (0.92)	2.38 (0.52)	4.17 (0.52)	3.61 (0.63)	3.04 (0.57)

*ES = emotion socialization.*

*^∗^*p* < 0.05. ^∗∗^*p* < 0.01. ^∗∗∗^*p* < 0.001. ^†^*p* < 0.07.*

Observer ratings of sensitive parenting composite was positively related to mother ratings of supportive emotion socialization and negatively related to non-supportive emotion socialization. To prevent common method variance in the regression analyses reported below, we aggregated observer ratings of sensitive parenting with mother ratings of supportive emotion socialization by standardizing and averaging these two variables. This composite variable was labeled as an *aggregated sensitive parenting score*. We kept the non-supportive dimension of emotion socialization as a separate score to investigate the role of self-construal on negatively controlling parenting. Finally, none of the demographic variables (i.e., child age and sex, mother age) was related to our parenting variables. Thus, these variables were not included in hierarchical regression models. The attentional control aspect of child temperament was only related to the aggregated sensitive parenting score, *r* = 0.35, *p* = 0.007.

### Testing the Unique Contribution of the Self-Construal to Parenting Practices

A total of two hierarchical multiple regression analyses were conducted to examine whether mothers’ autonomous-related self-construal made a significant, independent contribution to the prediction of parenting dimensions, after accounting for family SES and maternal efficacy. As seen in **Table 2**, the same order of entry was used for both sets of analyses, with SES and maternal efficacy entered in the first step, followed by the autonomous-related self-construal entered in the second step. The model with the aggregated sensitive parenting score also included child temperament in the first step as a control variable.

**Table 2:** Predicting parenting from SES, maternal efficacy, and self-construal.

		Sensitive Parenting				Negatively Controlling Parenting		
	*ΔR^2^*	*β*	*t*	*p*	*ΔR^2^*	*β*	*t*	*p*
**Step 1**	0.30				0.41			
SES		0.27	2.27^∗^	0.027		–0.46	–4.42^∗∗∗^	0.000
Maternal efficacy		0.26	2.16^∗^	0.035		–0.38	–3.66^∗∗∗^	0.001
**Step 2**	0.05				0.01			
SES		0.17	1.12	0.167		–0.45	–4.09^∗∗∗^	0.000
Maternal efficacy		0.23	1.91^†^	0.062		–0.38	–3.61^∗∗∗^	0.001
AR self-construal		0.26	2.08^∗^	0.043		–0.01	–0.13	0.901

*AR = autonomous-related. The model with sensitive parenting controlled for temperament.*

*^∗^*p* < 0.05. ^∗∗^*p* < 0.01. ^∗∗∗^*p* < 0.001. ^†^*p* < 0.10.*

#### Aggregated Sensitive Parenting Across Observer and Mother Ratings

The overall regression model with all predictors was significant, *F*(4,53) = 7.09, *p* < 0.001, and explained 35% of the variance. In the first step, both SES and maternal efficacy were significant predictors and accounted for 13% of the variance. Mothers from higher SES families and those with higher efficacy beliefs were more likely to enagage in sensitive parenting. In the second step, the addition of the autonomous-related self-construal explained an additional and unique 5% of the variance above and beyond SES, maternal efficacy, and child temperament, Δ*F*(1,53) = 4.32, *p* = 0.043.

#### Non-supportive Parenting

The overall regression model was significant, *F*(3,54) = 14.53, *p* < 0.001, explaining 45% of the variance. In the first step, both SES and maternal efficacy were significant predictors. Mothers from higher SES and those with a higher sense of efficacy were less likely to endorse non-supportive responses. The squared semipartial correlations as an index of the independent contribution to variance revealed that SES and maternal efficacy accounted for 19% and 13% unique variation, respectively. *R*^2^ change was not significant for entry of Step 2, suggesting that mothers’ autonomous-related self-construal did not explain additional variance.

#### Testing the Mediational Role of Mothers’ Autonomous-Related Self-Construal and Efficacy

Next, a multiple mediation model was conducted to simultaneously test indirect effects of mothers’ self-construal and efficacy beliefs between SES and sensitive parenting, controlling for child temperament. As noted by Preacher and Hayes (2008), a multiple mediation model yields an estimate of the total indirect effect, which represents an aggregated mediating effect of all mediators included in the analysis, and the specific indirect effects of each hypothesized mediator. We relied on empirical bootstrapping because this technique with a non-parametric resampling procedure is considered as a powerful way of testing indirect effects, especially with small samples (Preacher and Hayes, 2008). The PROCESS module estimated the coefficients for paths *a* and *b*, direct effect (path *c*) and indirect effect (path *c*) between the predictor and outcome variable, bootstrapped standard errors, and bias-corrected, accelerated 95% confidence intervals (BCa 95% CI). If the confidence interval does not contain zero, an effect is considered as significant.

The direct effect of SES on sensitive parenting composite (path *c*) was significant (*b* = 0.31, *p* = 0.006). SES was also significantly related to the two mediators (path *a*). SES had a positive relation with autonomous-related self (*b* = 0.22, *p* = 0.002), and with maternal efficacy (*b* = 0.15, *p* = 0.048). Sensitive parenting composite was also influenced by the self-construal in the presence of SES and maternal efficacy (path *b*). Specifically, mothers’ self-construal had a significant effect on sensitive parenting (*b* = 0.43, *p* = 0.043), whereas efficacy approached but did not reach significance (*b* = 0.36, *p* = 0.06). In the presence of the hypothesized mediators, the direct effect of SES on sensitive parenting (path *c*) was no longer significant (*b* = 0.16, *p* = 0.167). Examination of the confidence intervals indicated a significant total indirect path, *b* = 0.15, BCa 95% CI [0.031, 0.355], a marginally significant specific indirect effect through autonomous-related self-construal *b* = 0.09, BCa 95% CI [0.002, 0.275] while controlling for the indirect role of maternal efficacy, and a non-significant specific indirect effect through maternal efficacy *b* = 0.05, BCa 95% CI [-0.000, 0.194] while controlling for the indirect role of self-construal. In an exploratory analysis with mothers’ self-construal as the only mediator between SES and sensitive parenting, the indirect effect became significant, *b* = 0.11, BCa 95% CI [0.011, 0.312].

In the case of non-supportive parenting, hierarchical regression analyses suggested that SES remained a significant predictor of non-supportive parenting with mothers’ self-construal and efficacy in the model. Although the total effect parameter estimate of -0.33, BCa 95% CI [-0.461, -0.203] dropped to the direct effect parameter estimate of -0.27, BCa 95% CI [-0.402, -0.142], the confidence interval of both indirect effects contained zero, suggesting that partial mediation was not occurring either.

### Exploratory Analyses

Two additional hierarchical models were computed with the related self-construal as a predictor entered in step 2. Related self-construal did not explain incremental variance over and above family SES and maternal efficacy in the prediction of our two parenting variables.

## Discussion

Guided by the seminal parenting models (Belsky, 1984; Grolnick, 2002) and Kagitcibasi’s (2007) self models, the present study examined the relative contribution of Turkish mothers’ self-construal to parenting practices above and beyond family SES and maternal efficacy beliefs. Our findings revealed that family SES and maternal efficacy were two predictors of sensitive parenting based on the aggregated observer and mother ratings. Turkish mothers’ autonomous-related self-construal correlated positively with SES as expected and made a significant incremental contribution to the variance in sensitive parenting beyond the main effects of SES, maternal efficacy, and child temperament. In the case of mother ratings of non-supportive parenting, only family SES and maternal efficacy were significant predictors. Mothers’ autonomous-related self-construal did not contribute additional variance above and beyond these predictors. Finally, the specific indirect effect of the autonomous-related self between family SES and sensitive parenting was marginally significant when controlling for the indirect role of maternal efficacy. Below we discuss our significant and non-significant findings.

### Intracultural Variations in Mothers’ Self-Construal

Our first hypothesis was based on Kagitcibasi’s (2007) theoretical premise that individuals in urban, middle-high SES families in collectivist cultures would be more likely to construe an autonomous-related self view. In line with our prediction and consistent with the theory, Turkish mothers with higher SES background endorsed more autonomous-related self-characteristics than their lower SES counterparts. Unexpectedly, related self-construal was also positively associated with SES, suggesting the significance of social responsibilities and permeable self-boundaries in the definition of self among higher SES mothers. Yet, mothers’ related self-scores were not related to our parenting measures. Finally, as predicted, mothers’ autonomous-separate self-construal scores were neither related to SES nor to parenting.

This pattern of results suggested that rather than a transition from collecvtisitic to individualistic values in the face of modernization and economic development, younger and educated Turkish mothers view themselves as increasingly self-reliant yet related at the same time. As argued by Kagitcibasi (2007), within the context of rapid social change in collectivist societies, embracing an autonomous-related self-construal may render more adaptive to the changing lifestyle of younger generations. Our finding also extended existing research by documenting the compatibility of autonomy and relatedness not only in mothers’ child socialization goals (Mayer et al., 2012), but also in the self-construal of educated mothers in developing collectivist societies.

### Role of the Autonomous-Related Self-Construal on Sensitive Parenting

Our next question concerned the relationship between mothers’ self-construal and parenting practices over and above the social-contextual and parent characteristics commonly associated with child socialization. Based on previous research, we considered maternal efficacy as a maternal personal resource (Jones and Prinz, 2005), and SES as a family resource that commonly represents the financial capital as well as education-related knowledge and skills to promote developmentally-facilitating interactions in the family (Bornstein and Bradley, 2002). As cultural and intracultural values influence how the self is conceptualized (Kagitcibasi, 2007; Markus and Kitayama, 2010), and one’s view about the nature of self may be reflected in child rearing patterns (Dennis et al., 2002; Trommsdorff and Cole, 2011), we expected that mothers’ autonomous-related self-construal would make an additional contribution to parenting beyond family SES and maternal efficacy.

In the present study, sensitive parenting was conceptualized as encompassing mothers’ display of warmth, responsiveness to the child’s cues, and autonomy granting, as aggregated across observer ratings in three dyadic contexts in the laboratory setting as well as mother ratings of emotion socialization. Mothers’ behavioral and affective qualities cohered together meaningfully around this construct as documented in previous research (Carlson et al., 2004; Jaffari-Bimmel et al., 2006; Burchinal et al., 2014). In the present study, mother ratings of child’s temperament were also related to the sensitive parenting aggregate; thus, child temperament was controlled when testing hypotheses about this dimension of parenting.

In line with our predictons, Turkish mothers’ autonomous-related self-construal explained additional variance in our aggregated sensitive parenting variable over and above the main effects of family SES, maternal efficacy, and child temperament. Turkish mothers, who endorsed a more balanced combination of relatedness and autonomy for the nature of their selves, were more likely to combine autonomy granting with warmth and responsiveness in their interactions. The assessment of sensitive parenting in the present study that combined behavioral observations with mother ratings reduces the possibility that shared method variance accounted for this relation.

Moreover, lending support to Belsky’s model of parenting, our findings also revealed that maternal efficacy and family SES predicted sensitive parenting as expected. Mothers who perceived themselves as efficacious in caregiving and those from higher SES families were more likely to have an affectively positive tone in their interactions and responded appropriately to their child’s needs while following the child’s lead whenever possible. These findings were consistent with past research (Zevalkink and Riksen-Walraven, 2001; Ispa et al., 2004; Jones and Prinz, 2005). Previous research with Turkish mothers of 3-year-old children also showed a similar SES pattern in autonomy-supporting and responsive parenting during home observations (Akcinar and Baydar, 2014; Baydar and Akcinar, 2015). Our results extended past research by replicating these SES differences in a laboratory setting with Turkish mothers of preschool-aged children and by investigating the additive effects of SES with maternal efficacy on parenting as well.

We also examined whether autonomous-related self-construal and maternal efficacy would both simultaneously act as potential explanatory mechanisms between family SES and sensitive parenting. Our results revealed that only the specific indirect effect through mothers’ self-construal approached significance, while controlling for the indirect effect of maternal efficacy. Insufficient statistical power may be an explanation for this statistically suggestive effect with our sample of 58 mothers considering the small effect sizes for self-construal in previous research with adults (Cross et al., 2011). We also conducted a conservative test of the indirect role of self-construal since we have controlled for the indirect role of maternal efficacy in the same model. Indeed, when maternal efficacy was excluded from the mediational model in an exploratory analysis, the indirect effect from SES to sensitive parenting via autonomous-related self-construal became significant. While the mediating effect of mothers’ self-construal detected in the present study needs to be replicated in future research, this finding was in line with theory (Kagitcibasi, 2007) and suggested that families’ sociodemographic features related to mothers’ balanced view of relatedness and autonomy of self, which in return predicted sensitive parenting that combined mothers’ responsiveness and autonomy granting. Finally, given the small effect sizes, rather than mothers’ general perspective about themselves, culturally-mediated yet more domain-specific beliefs may better explain intracultural differences in parenting. For example, mothers’ self view may organize how they would define a socially and emotionally competent child, which in turn would shape their parenting practices (Trommsdorff et al., 2012). Assessment of such ethnotheories are important for future studies to facilitate the interpretation of intracultural differences in parenting.

### Role of the Autonomous-Related Self-Construal on Non-supportive Parenting

The second parenting composite in the present study consisted of mother ratings of punishing, overriding and distress magnifying responses to child’s negative emotions. This composite reflected a non-supportive approach, consistent with previous research with typical Western samples (Eisenberg et al., 1999). Such negatively controlling responses involve communicating high negative appraisal of the child’s self, and such disapproval may threaten child’s self-esteem and autonomy (Kagitcibasi, 2007). Thus, we expected mothers’ autonomous-related self-construal to contribute to less non-supportive responses.

Although bivariate relations demonstrated an inverse relation between the autonomous-related self-construal and non-supportive parenting, mothers’ self-construal did not contribute to this parenting dimension above and beyond family SES and maternal efficacy. This suggested that mothers’ domain-specific competency beliefs in caregiving along with family SES played a relatively more important role, consistent with previous research (Jones and Prinz, 2005). These findings also suggested that feelings of inadequacy and loss of control during mother–child interactions may act to increase mothers’ negatively controlling and coercive behaviors, possibly in order to reestablish the perceived loss of power-balance in their relationship.

In the present study, SES had more predictive power than maternal efficacy on non-supportive parenting, suggested by the semipartial correlations of unique variation explained. Previous research has also established the pivotal role of family SES and its components, particularly maternal education that supports mothers’ knowledge about child development and parenting skills (Bornstein and Bradley, 2002). Mothers’ cognitive competency may also foster increased motivation to act as non-aggressive models for their children in the context of emotionally-charged situations.

Our results have also revealed that neither the autonomous-related self-construal nor maternal efficacy mediated the relation between family SES and non-supportive parenting. As previous research suggests, the primary mechanism by which socioeconomic disadvantage fosters negatively controlling parenting seems to be through caregivers’ mental health. Low SES has been associated with poorer caregiver mental health and emotional well-being as well as family dysfunction, which in return increase hostility displayed toward children (Conger et al., 2000).

### Limitations

A major strength of this study was its use of behavioral observations of mother–child interactions during three dyadic contexts that involved free play, snack time, and a drawing activity in the laboratory setting. We also included positive affect displays during snack time as an additional indicator of maternal warmth. Furthermore, we combined our assessment of sensitive parenting based on observational data with mother ratings of emotion socialization to obtain a more comprehensive picture of parenting. Research indicates that aggregated data provide a more stable and representative picture of caregiving than do data from single method and informants (Wachs, 1987). However, a potential limitation of the present study was the measurement of non-supportive parenting, solely drawn from mother ratings. This might have attenuated assessment accuracy and impacted the pattern of findings. Yet, even when assessed with mother ratings only, significant bivariate relations between this parenting dimension and SES as well as maternal efficacy were detected, largely consistent with previous literature. For multiple mediation analyses, our sample size was not large enough to detect statistically significant indirect effects with small effect sizes, and therefore lacked statistical power. Finally, our assessment of children’s temperament was limited to the attentional regulation component and lacked the scales that tap emotional reactivity. Finally, our study participants were from one major metropolitan area in Turkey. Whether the results would generalize to Turkish mothers who live in other urban areas remains to be investigated.

## Conclusion

Our findings have shown that, with increasing education and affluence level, Turkish mothers were more likely to endorse the compatibility of autonomy with relatedness as a way of being and and relating to others. This study also provided valuable information about the relative contribution of mothers’ self-construal beyond family SES and maternal efficacy in predicting parenting. Sensitive parenting that combined warmth, responsiveness and autonomy granting was uniquely predicted by mothers’ autonomous-related self-construal. In the prediction of negatively controlling parenting, family SES and domain-specific factors at the individual level (i.e., maternal efficacy) emerged as significant predictors. Finally, our findings suggest new directions for future research to better understand how mothers convey the nature of their self views in socializing their children. How mothers’ general perspective about themselves translates into more specific, intuitive ethnotheories of competent children remains to be investigated for a better understanding of the cultural models of self and child socialization.

## Ethics Statement

This study was carried out in accordance with the recommendations of Declaration of Helsinki guidelines, Boğaziçi University Institutional Review Board for Research with Human Subjects with written informed consent from all subjects. All subjects gave written informed consent in accordance with the Declaration of Helsinki. The protocol was approved by the Boğaziçi University Institutional Review Board for Research with Human Subjects.

## Author Contributions

FC formulated the research hypotheses and study design. She trained all observers to code maternal behaviors and completed data analyses. She was also the lead author of the submitted manuscipt and completed most of the writing. HB was responsible for subject recruitment and for conducting all the behavioral assessments in the research laboratory. She also monitored and supervised the coders. SB assisted with the literature review of the research topic and contributed to the writing up of the manuscipt. She was also involved in the data collection and data analyses.

## Conflict of Interest Statement

The authors declare that the research was conducted in the absence of any commercial or financial relationships that could be construed as a potential conflict of interest.
